# *HHEX_23* AA Genotype Exacerbates Effect of Diabetes on Dementia and Alzheimer Disease: A Population-Based Longitudinal Study

**DOI:** 10.1371/journal.pmed.1001853

**Published:** 2015-07-14

**Authors:** Wei-Li Xu, Nancy L. Pedersen, Lina Keller, Grégoria Kalpouzos, Hui-Xin Wang, Caroline Graff, Bengt Winblad, Lars Bäckman, Laura Fratiglioni

**Affiliations:** 1 Aging Research Center, Department of Neurobiology, Care Sciences and Society, Karolinska Institutet and Stockholm University, Stockholm, Sweden; 2 Department of Epidemiology and Biostatistics, School of Public Health, Tianjin Medical University, Tianjin, China; 3 Department of Medical Epidemiology and Biostatistics, Karolinska Institutet, Stockholm, Sweden; 4 Alzheimer’s Disease Research Center, Department of Neurobiology, Care Sciences and Society, Karolinska Institutet, Stockholm, Sweden; 5 Department of Geriatric Medicine, Karolinska University Hospital, Stockholm, Sweden; 6 Stockholm Gerontology Research Center, Stockholm, Sweden; Mount Sinai School of Medicine, United States

## Abstract

**Background:**

Research has suggested that variations within the *IDE/HHEX* gene region may underlie the association of type 2 diabetes with Alzheimer disease (AD). We sought to explore whether *IDE* genes play a role in the association of diabetes with dementia, AD, and structural brain changes using data from two community-based cohorts of older adults and a subsample with structural MRI.

**Methods and Findings:**

The first cohort, which included dementia-free adults aged ≥75 y (*n* = 970) at baseline, was followed for 9 y to detect incident dementia (*n* = 358) and AD (*n* = 271) cases. The second cohort (for replication), which included 2,060 dementia-free participants aged ≥60 y at baseline, was followed for 6 y to identify incident dementia (*n* = 166) and AD (*n* = 121) cases. A subsample (*n* = 338) of dementia-free participants from the second cohort underwent MRI. *HHEX_23* and *IDE_9* were genotyped, and diabetes (here including type 2 diabetes and prediabetes) was assessed. In the first cohort, diabetes led to an adjusted hazard ratio (HR) of 1.73 (95% CI 1.19–2.32) and 1.66 (95% CI 1.06–2.40) for dementia and AD, respectively, among all participants. Compared to people carrying the GG genotype without diabetes, AA genotype carriers with diabetes had an adjusted HR of 5.54 (95% CI 2.40–7.18) and 4.81 (95% CI 1.88–8.50) for dementia and AD, respectively. There was a significant interaction between *HHEX_23*-AA and diabetes on dementia (HR 4.79, 95% CI 1.63–8.90, *p* = 0.013) and AD (HR 3.55, 95% CI 1.45–9.91, *p* = 0.025) compared to the GG genotype without diabetes. In the second cohort, the HRs were 1.68 (95% CI 1.04–2.99) and 1.64 (1.02–2.33) for the diabetes–AD and dementia–AD associations, respectively, and 4.06 (95% CI 1.06–7.58, *p* = 0.039) and 3.29 (95% CI 1.02–8.33, *p* = 0.044) for the interactions, respectively. MRI data showed that *HHEX_23*-AA carriers with diabetes had significant structural brain changes compared to *HHEX_23*-GG carriers without diabetes. No joint effects of *IDE_9* and diabetes on dementia were shown. As a limitation, the sample sizes were small for certain subgroups.

**Conclusions:**

A variant in the *HHEX_23* gene interacts with diabetes to be associated with a substantially increased risk of dementia and AD, and with structural brain changes among dementia-free elderly people.

## Abstract 2

In a longitudinal study, Weili Xu and colleagues explore whether variants in two insulin pathway genes modify the association of type 2 diabetes with dementia and AD.

## Editors' Summary

**Background:**

Worldwide, about 44 million people have dementia, a group of degenerative, incurable brain disorders that mainly affect older people. Dementia is characterized by an irreversible decline in memory, communication, and other “cognitive” functions. The most common forms of dementia are Alzheimer disease, which is caused by the development of small clumps of proteins (β-amyloid plaques) around brain cells, and vascular dementia, which is caused by reduced blood flow to parts of the brain. Early symptoms of dementia include increasing forgetfulness and losing track of time. As the condition progresses, affected individuals gradually lose the ability to look after themselves and to communicate, and they may become anxious or aggressive. Eventually, affected individuals may lose control of various physical functions and many become totally dependent on specialist nurses and other professional carers for their day-to-day needs.

**Why Was This Study Done?:**

Epidemiological studies (investigations that examine patterns of disease in populations) suggest that, among older individuals, having type 2 diabetes (a condition in which resistance to the hormone insulin leads to high blood sugar levels) is associated with a 50% increased risk of developing dementia. Even prediabetes (a blood sugar level that is high but not high enough to meet the criteria for diabetes) is associated with an increased risk of dementia. Prediabetes and diabetes increase the risk of vascular disease, but it is thought that polymorphisms (naturally occurring genetic variations) in the *IDE/HHEX* region of the human genome may be involved in the association between diabetes and dementia/Alzheimer disease. *IDE* encodes insulin-degrading enzyme, which clears insulin from cells but also degrades β-amyloid; *HHEX* encodes a transcription factor that controls *IDE* expression. In this population-based longitudinal study, the researchers explore whether two single nucleotide polymorphisms in this region—*IDE_9* and *HHEX_23*—impact the association between diabetes and dementia. Population-based longitudinal studies measure the baseline characteristics of individuals in the general population and determine which individuals subsequently develop specific conditions.

**What Did the Researchers Do and Find?:**

For their study, the researchers examined data collected from two cohorts (groups) of dementia-free elderly adults living in Stockholm, Sweden. Blood samples taken from 3,030 participants at baseline were used to determine which individuals had diabetes (type 2 diabetes or prediabetes) and to investigate which *HHEX_23* and *IDE_9* variants each individual carried (genotyping). The researchers also examined the brain structure of a subsample of the second cohort using magnetic resonance imaging (MRI). All the participants were followed for several years to see which individuals developed dementia/Alzheimer disease. In both cohorts, having diabetes was associated with a 60% increased risk of dementia/Alzheimer disease after adjusting for other characteristics that affect the development of these conditions. Pooled data from both cohorts indicated that, compared to people without diabetes carrying *HHEX_23*-GG (the human genome contains two copies [alleles] of each gene, and an individual carrying *HHEX_23*-GG has the nucleotide guanine at a particular position in both *HHEX_23* copies), people with diabetes carrying *HHEX_23*-AA (adenine at that position in both *HHEX_23* copies) had a 5-fold higher risk of developing dementia/Alzheimer disease. Other analyses indicated that the *HHEX_23*-AA genotype interacted with diabetes to substantially increase the risk of dementia/Alzheimer disease. Finally, MRI showed that, at baseline, *HHEX_23*-AA carriers with diabetes had structural brain changes compared to *HHEX_23*-GG carriers without diabetes, even though they were all free of dementia at the time.

**What Do These Findings Mean?:**

These and other findings indicate that polymorphisms in *HHEX_23*, but not *IDE_9*, are involved in the association between diabetes, dementia, and structural brain changes. Thus, a genetic variant in *HHEX_23* may play an important role in the development of Alzheimer disease and other forms of dementia among people with diabetes or prediabetes. These findings may not apply to younger or rural populations, and their accuracy may be affected by the use of a single blood sugar level reading to diagnose diabetes and prediabetes. Moreover, because some of the genotype/diabetes subgroups contained very few people, these findings need to be confirmed in additional studies. Further studies are also needed to understand the role of *HHEX_23* in the association between diabetes and dementia. Importantly, however, these findings highlight the need to control diabetes to prevent the development of Alzheimer disease and other forms of dementia, particularly among *HHEX_23*-AA carriers.

**Additional Information:**

This list of resources contains links that can be accessed when viewing the PDF on a device or via the online version of the article at http://dx.doi.org/10.1371/journal.pmed.1001828.

- The UK National Health Service Choices website provides information (including personal stories and links to additional resources) about dementia, vascular dementia, Alzheimer disease, and diabetes
- The UK not-for-profit organization Alzheimer’s Society provides information for patients and carers about dementia, including personal experiences of dementia
- The US not-for-profit organization Alzheimer’s Association also provides information for patients and carers about dementia, personal stories about dementia, and information about the association between diabetes and dementia
- Alzheimer’s Disease International is the federation of Alzheimer disease associations around the world; it provides links to individual Alzheimer disease associations, information about dementia, and links to World Alzheimer Reports
- MedlinePlus provides links to additional resources about dementia, Alzheimer disease, vascular dementia, and diabetes (in English and Spanish)
- More information about the Kungsholmen Project and the Swedish National Study on Aging and Care–Kungsholmen, the projects that enrolled the cohorts used by the researchers for this study, is available

## Introduction

An increased risk of dementia and Alzheimer disease (AD) in people with type 2 diabetes has been found in numerous prospective population-based studies, including several meta-analyses using pooled data [1–3]. Older individuals with diabetes have on average a 50% greater risk of dementia compared to those without diabetes [2]. Even prediabetes has been found to be predictive of an increased risk of dementia and AD [4,5]. However, mechanisms for such an association remain unclear. In addition to vascular mechanisms, genetic background has been implicated in AD related to diabetes [6].

Insulin degrading enzyme (IDE) is the primary enzyme responsible for insulin clearance in cells. Changes in insulin metabolism are fundamental to insulin resistance. The gene encoding IDE is located on Chromosome 10 q23-q25 in humans, a region that includes *IDE* and *HHEX* (homeobox, hematopoietically expressed) [7]. The protease function of IDE is mediated by the exons of *IDE*, and the regulation of *IDE* gene expression is likely buried in the surrounding sequences of exons [7]. Overexpression of *IDE* in cells has been found to increase insulin degradation [8]. On the other hand, the injection of *IDE-*specific antibodies into cells inhibits the process of insulin degradation [9]. Variations in *HHEX*_23 may influence the expression of *IDE* [10].

IDE has also been shown to effectively degrade different forms of β-amyloid (Aβ) [7], which is involved in AD pathology. Linkage and association studies have shown evidence for an involvement of sequences near to the *IDE* region in AD [11]. Thus, *IDE/HHEX* genes could be candidate genes for both type 2 diabetes and AD [7]. Indeed, *IDE/HHEX* genes have been linked to type 2 diabetes and elevated blood glucose level [12,13]. Although genetic association studies on SNPs in the *IDE/HHEX* region and AD have produced both positive and negative findings [14], several studies have suggested that genetic variations within the *IDE/HHEX* gene region may impact both diseases, and thus underlie the association between diabetes and AD [7]. So far, no studies have examined the role of *IDE/HHEX* genes in such an association in humans.

Within the Kungsholmen Project (KP), we have previously reported an increased risk of dementia and AD in people with diabetes or prediabetes [4,15–18]. In a large population-based study of Swedish twins, we further observed that genetic background contributed to the diabetes–dementia association [18]. On the basis of these findings and other reports, we genotyped two SNPs located in the 276-kb haplotype block including the “tag” SNPs of *HHEX*_23 (rs1544210) and *IDE*_9 (rs1887922) that were previously reported to be associated with AD [11,19]. The prevalence of the A (G) allele of *HHEX_23* and the T (C) allele of *IDE*_9 is 50.7% (49.4%) and 82.5% (17.4%), respectively, in the Swedish population [11,19] and 47%–51% (49%–53%) and 82%–84% (16%–18%) in the American and English populations [20], respectively. In the present study, we aimed to explore whether variations in *HHEX*_23 and *IDE*_9 play a role in the association of diabetes with dementia and AD using data from two population-based longitudinal studies and a structural MRI study.

## Methods

### Study Populations

#### The Kungsholmen Project

The KP is a community-based prospective study on aging and dementia [21]. All registered inhabitants who were living in the Kungsholmen district of Stockholm, Sweden, and were aged ≥75 y on October 1, 1987, were initially invited to participate in the project. At baseline (1987–1989), a two-phase survey that consisted of a screening phase and a clinical phase was implemented to identify prevalent dementia cases. The screening phase included a health interview and administration of the Mini-Mental State Examination (MMSE) in all participants. In the clinical phase, all participants who screened positive for impaired cognitive function (MMSE score ≤ 23) were invited to undertake a comprehensive physical, neurological, and psychiatric examination. After the baseline survey (screening and clinical phase), 225 of 1,700 participants were diagnosed as having prevalent dementia according to the criteria of the *Diagnostic and Statistical Manual of Mental Disorders*, *Third Edition–Revised* (*DSM-III-R*) [22], leaving 1,475 participants for the dementia-free cohort. Of them, two participants had mental disorders, and 172 refused to participate in the first follow-up examination (1991–1993) or had moved [16]. Of the remaining 1,301 dementia-free participants, 970 participants were left for the current study after excluding two individuals with type 1 diabetes and 329 with no blood samples or poor quality of DNA extraction.

During a 9-y follow-up, three clinical examinations were carried out at an average interval of 3 y until 1997–1998. Throughout the follow-up period, of the 970 participants, 346 participants died and 51 did not participate in the second or third follow-up exams.

#### The Swedish National Study on Aging and Care–Kungsholmen

The Swedish National Study on Aging and Care–Kungsholmen (SNAC-K) is a part of the Swedish National Study on Aging and Care, an ongoing longitudinal project focusing on the aging process and the Swedish care system [23]. The study population consisted of a random sample of all registered inhabitants who were aged ≥60 y living in the Kungsholmen district of Stockholm in March 2001. The sample was stratified by age cohort and year of assessment because of the more rapid changes in health and higher attrition rate among older age groups. In total, 11 age-specific cohorts were chosen with different time intervals of assessment: a 6-y interval in young cohorts (60, 66, and 72 y) and a 3-y interval in older cohorts (78, 81, 84, 87, 90, 93, 96, and ≥99 y). At baseline (2001–2004), among the 4,590 eligible persons, 3,363 (73.3%) participants were examined. Of them, 250 were diagnosed as having prevalent dementia (*DSM-III-R*), and 3,113 were identified as dementia-free after baseline examination. Of the dementia-free participants, 2,060 were left for the current analysis after excluding five individuals with type 1 diabetes and 1,048 individuals without blood samples or with poor quality of DNA extraction. During the 6-y follow-up period (until 2007–2008), of the 2,060 participants, 588 participants died and 76 refused participation.

#### SNAC-K MRI study

The MRI sample was a subpopulation of non-disabled and non-demented participants from SNAC-K measured during 2001–2004. In total, 552 consecutive participants received structural MRI scans on a 1.5T scanner (Philips Intera). Of them, 338 participants were included in the analysis after excluding participants with neurological disease (*n* = 20), suboptimal MRI quality (*n* = 9), or no genotyping data available (*n* = 185).

Written informed consent was received from all participants or from informants when the person was cognitively impaired. The Ethics Committee at the Karolinska Institutet approved all phases of the KP, the SNAC-K, and the MRI study.

### Data Collection

In both the KP and SNAC-K, data on age, sex, and education were collected from participants at baseline following standardized protocols [20,23]. Education was measured as number of years of formal schooling and was dichotomized (≥8 versus <8 y) [24]. Global cognitive functioning was assessed with the MMSE. Weight and height were measured with a standard scale in light clothing and with no shoes, and body mass index (BMI) was calculated as weight (in kilograms) divided by the square of height (in meters). Arterial blood pressure (i.e., systolic Korotkoff phase I and diastolic phase V) was measured on the right arm with the participant in a sitting position after at least a 5-min rest.

Information on medical history was taken from the inpatient register system [25]. The International Classification of Diseases (ICD-8, ICD-9, and ICD-10) has been used in the system since 1969. Medical characteristics—including BMI, systolic blood pressure (SBP), diastolic blood pressure (DBP), heart disease (ischemic heart disease, heart failure or other myocardial insufficiency, and atrial fibrillation), stroke, and type 2 diabetes—were derived from the inpatient register database and clinical examinations. Medical drugs were coded according to the Anatomical Therapeutic Chemical Classification System [26]. Hypoglycemic drugs included hypoglycemic medications or insulin injection (ATC code A10).

### Assessment of Type 2 Diabetes and Prediabetes

Blood samples were taken at the baseline survey. Random blood glucose was measured using a glucose oxidase procedure [27]. Type 2 diabetes was ascertained through medical examinations, inpatient register (ICD-8 and ICD-9 code: 250; ICD-10 codes E11–E14), use of hypoglycemic drugs (ACT code A10), or random blood glucose level ≥ 11.0 mmol/l [16,28]. Prediabetes was defined as a random blood glucose concentration of 7.8–10.99 mmol/l among participants not already diagnosed with diabetes [16,29].

### SNP Selection and Genotyping

Genomic DNA was extracted from whole blood samples. We genotyped two markers including *HHEX*_23 (rs1544210) and *IDE*_9 (rs1887922) from the region surrounding *IDE* and spanning a 276-kb linkage disequilibrium block. These two markers were previously identified from 26 markers in the 480-kb region surrounding *IDE* and were found to be associated with AD by Prince et al. [11] (with linkage disequilibrium metrics) and Björk et al. [19] (based on several independent sets of the Swedish case–control analyses). Within this region, three SNPs—rs2251101 (*IDE_7*), rs12783634, and rs1999763—were additionally genotyped based on another report showing their possible association with AD in other populations [30].

Genotyping of SNPs was performed using matrix adsorbed laser desorption ionization–time of flight analysis on a Sequenom MassARRAY platform. To verify the polymorphisms of the SNPs, each was tested in a set of 16 Swedish control samples. *APOE* allelic status was determined using a microsequencing method (AffiGen APOE, Sangtec Medical) based on a polymerase chain reaction with biotinylated primers. The genotyped SNPs can be found at dbSNP (http://www.ncbi.nlm.nih.gov/SNP/). SNP genotyping success rate was >95%. For quality control of SNP genotyping, each 96-well plate contained three or more duplicate samples and a negative control. The concordance rate for genotyping was >99.5%.

### Dementia and Alzheimer Disease Diagnosis

In both projects, at each follow-up, all participants underwent a comprehensive clinical examination and cognitive tests. Dementia was diagnosed following the *DSM-III-R* criteria, in which a validated three-step diagnostic procedure was used as previously reported [31]. In brief, two examining physicians independently made a preliminary diagnosis, and in the case of disagreement, a third opinion was sought to reach a consensus diagnosis. The diagnosis of AD required gradual onset, progressive deterioration, and lack of any other specific causes of dementia, according to the National Institute of Neurological and Communicative Disorders and Stroke–Alzheimer’s Disease and Related Disorders Association (*NINCDS-ADRDA*) criteria [32]. For the deceased participants, the diagnosis of dementia and its subtypes was made by two physicians through reviewing the medical records and death certificates. The physicians did not have access to the genetic data.

### MRI Acquisition and Reading Protocol

The MRI protocol included an axial 3-D T1-weighted fast field echo, an axial proton density/T2 turbo spin echo run twice with a shift of 3 mm, and an axial turbo fluid-attenuated inversion recovery (FLAIR) sequence [33]. Total gray matter volume (GMV) and white matter volume (WMV) were calculated after automatic segmentation of the T1 images in native space using SPM12b software (http://www.fil.ion.ucl.ac.uk/spm/) implemented in Matlab (MathWorks) [34]. All gray matter, white matter, and cerebral spinal fluid (CSF) segments were individually scrutinized for verification of the segmentation accuracy. Hippocampal and lateral ventricular volumes were manually delineated on the T1 images following procedures described previously [35]. White matter hyperintensity (WMH) volumes were manually delineated on the FLAIR images. Intracranial volume (ICV) was obtained by summing up volumes of gray matter, white matter, and CSF. ICV was used to adjust the regional volumes [36], and the adjusted volumes were used in the following analysis.

### Statistical Analysis

Statistical differences were examined with chi-square tests for categorical variables and one-way ANOVA for continuous variables, with post hoc comparison. Bonferroni corrections were used for multiple comparisons. Hardy–Weinberg equilibrium for individual loci was assessed using the Pearson chi-square statistic. Logistic regression was used to estimate the odds ratio (OR) and 95% confidence interval for the relation of diabetes to the *IDE* genes. Cox proportional hazards models were used to estimate the hazard ratio (HR) and 95% CI of incident dementia and AD. For non-demented participants, the follow-up time was calculated from the date of the baseline interview to the date of the last follow-up examination or death. For demented participants, the follow-up time was estimated as the time during which participants were free of dementia plus half of the follow-up time during which dementia developed, because of its insidious onset. The proportional hazards assumption was confirmed by graphs and tests based on Schoenfeld residuals, which showed that the hazards were generally proportional over the follow-up period among groups of participants by diabetes status and genotype of the *IDE* genes. The combined effect of two factors was assessed by creating dummy variables based on joint exposures to both factors. We examined statistical interaction by incorporating the two variables and their cross-product term in the same model. Age, sex, education, MMSE score, BMI, heart disease, stroke, SBP, DBP, *APOE* ε4, and follow-up survival status were considered as potential confounders. All dementia (including AD) and AD were used as separate outcomes in Cox and logistic regression analyses. Kaplan–Meier survival analysis was used to compare the cumulative probability of events among participants in different groups.

Multiple imputation by chained equation was used for missing values for blood glucose and genotypes, with 20 completed datasets generated. Age, sex, education, BMI, heart disease, stroke, SBP, DBP, MMSE score, and *APOE* ε4 were considered as covariates in the multiple imputation.

In the MRI data analysis, linear regression was performed to estimate β coefficients (95% CIs) for diabetes by *IDE* gene in relation to regional brain volumes, CSF volume, and WMH. All statistical analyses were performed using Stata SE 12.0 for Windows (StataCorp) and SPSS Statistics for Windows, version 22.0 (IBM).

## Results

### Population Characteristics

In the KP, 82 (8.4%) participants had diabetes and 32 (3.3%) had prediabetes among the 970 dementia-free participants at baseline. The prevalence of diabetes including prediabetes was 11.7% (114/970), which is comparable with that in an elderly Swedish population in 1987 [37]. In the SNAC-K, the prevalence of diabetes including prediabetes was 35.1% (725/2,060), which is similar to that in a Swedish population aged ≥60 y in 2001 [38].

The differences between observed and expected frequencies of *HHEX_23* and *IDE_9* genotypes were not significant, and Hardy–Weinberg equilibrium was met in both cohorts. The A allele of *HHEX_23* was present in 50.6% and 50.7% of the participants in the KP and SNAC-K, respectively. The G allele was carried by 49.4% and 49.3% of the KP and SNAC-K populations, respectively. These frequencies are similar to those in the American English populations [20].

As the effect of diabetes and prediabetes on dementia and AD was similar to that shown in our previous studies [4,16], type 2 diabetes and prediabetes were combined together as “diabetes” in the following analysis. Characteristics of participants with different genotypes of *HHEX_23* and *IDE_9* were similar, except for the distribution of *HHEX_23* variants with regard to education in the SNAC-K and the distribution of *HHEX_23* variants with regard to diabetes in both cohorts (Table 1).

**Table 1:** Characteristics of the study populations from the Kungsholmen Project (*n* = 970) and the SNAC-K (*n* = 2,060) by *HHEX_23* and *IDE_9* genotype.

Characteristic	*HHEX_23* Genotype			*p-*Value	*IDE_9* Genotype			*p-*Value
	GG	AG	AA		TT	TC	CC	
**Kungsholmen Project**	250	458	262	0.084^a^	674	272	24	0.577^a^
Age (years)	80.5 (4.7)	80.6 (4.5)	80.5 (4.9)	0.914	80.5 (4.7)	80.7 (4.7)	79.2 (4.1)	0.353
Female sex	191 (76.4%)	329 (71.8%)	202 (74.9%)	0.410	493 (73.1%)	209 (76.8%)	19 (79.2%)	0.430
Educational level ≥ 8 y	103 (41.2%)	187 (40.8%)	121 (46.2%)	0.371	272 (40.5%)	127 (46.9%)	12 (50.0%)	0.156
MMSE score	26.7 (2.9)	26.9 (2.2)	26.9 (2.4)	0.720	26.9 (2.5)	26.8 (2.3)	27.1 (1.9)	0.777
Diabetes/prediabetes	26 (10.4%)	50 (10.9%)	38 (14.5%)	0.042	74 (10.9%)	37 (13.6%)	3 (12.5%)	0.730
BMI (kg/m^2^)	23.7 (3.8)	23.7 (3.3)	23.9 (3.5)	0.565	23.7 (2.9)	23.6 (3.0)	23.8 (3.1)	0.925
Any *APOE* ε4	78 (31.2%)	119 (26.0%)	67 (26.9%)	0.364	193 (30.0%)	67 (28.3%)	5 (20.8%)	0.267
**SNAC-K**	502	1024	534	0.799^a^	1,408	586	66	0.599^a^
Age (years)	75.4 (10.4)	75.6 (10.6)	75.9 (10.4)	0.714	75.8 (10.4)	75.2 (10.5)	76.1 (10.7)	0.540
Female sex	327 (65.1%)	663 (64.7%)	326 (61.0%)	0.282	908 (64.5%)	369 (63.0%)	39 (59.1%)	0.579
Educational level ≥ 8 y	427 (85.1%)	818 (80.0%)	451 (84.5%)	0.017	1,168 (83.0%)	471 (80.5%)	57 (86.4%)	0.294
MMSE score	28.3 (3.8)	28.1 (3.4)	28.2 (3.8)	0.652	28.1 (3.6)	28.3 (3.3)	27.9 (5.4)	0.092
Diabetes/prediabetes	167 (33.3%)	360 (35.2%)	198 (37.1%)	0.067	483 (34.3%)	214 (36.5%)	28 (42.4%)	0.293
BMI (kg/m^2^)	25.2 (3.7)	25.4 (3.9)	25.3 (3.9)	0.795	25.4 (3.9)	25.2 (3.6)	25.5 (4.5)	0.810
Any *APOE* ε4	140 (28.2%)	272 (27.1%)	161 (30.6%)	0.361	186 (25.1%)	80 (26.7%)	19 (29.7%)	0.864

Data are mean (standard deviation), number, or number (percentage) of participants.

^a^ *p*-Value for Hardy–Weinberg equilibrium.

Compared to participants with complete data, dropouts and participants with no blood samples for genotyping were older (81.0 versus 80.5 y, *p* < 0.05, in the KP, and 83.9 versus 83.4 y, *p* < 0.05, in the SNAC-K) and had lower MMSE scores (26.7 versus 26.9, *p* = 0.049, in the KP, and 27.1 versus 27.3, *p* = 0.042, in the SNAC-K), but there were no significant differences in terms of gender, education, BMI, heart disease, stroke, SBP, DBP, or diabetes between these groups in either study population.

### *HHEX_23* in Relation to Diabetes and Dementia

In crude and multi-adjusted (age, sex, education, stroke, heart disease, SBP, DBP, BMI, and *APOE* ε4) logistic regression analysis, compared to the *HHEX_23-*GG genotype, the *HHEX_23-*AG genotype (adjusted OR 2.36, 95% CI 1.04–5.35 in the KP, and OR 2.13, 95% CI 1.16–3.97, in the SNAC-K) and the AA genotype (adjusted OR 2.41, 95% CI 1.01–5.78, in the KP, and OR 2.36, 95% CI 1.28–3.96, in the SNAC-K) were significantly associated with diabetes in the two cohorts (Table 2). Using the *IDE_9*-TT as referent group, the TC genotype (adjusted OR 1.08, 95% CI 0.65–1.87, in the KP, and OR 1.18, 95% CI 0.66–3.03, in the SNAC-K) and the CC genotype (adjusted OR 0.84, 95% CI 0.56–2.66, in the KP, and OR 0.78, 95% CI 0.70–3.21, in the SNAC-K) were not significantly associated with diabetes (Table 2).

**Table 2:** Crude and multi-adjusted odds ratios and 95% CIs of the relation of diabetes and prediabetes to *HHEX_23* and *IDE_9* in the Kungsholmen Project and SNAC-K.

Genotype/Phenotype	Kungsholmen Project			SNAC-K		
	*n* ^a^	Diabetes/Prediabetes		*n* ^a^	Diabetes/Prediabetes	
		Crude OR (95% CI)	Adjusted OR (95% CI)^b^		Crude OR (95% CI)	Adjusted OR (95% CI) ^b^
***HHEX_23* genotype**						
GG	26/224	1.00 (Ref.)	1.00 (Ref.)	167/335	1.00 (Ref.)	1.00 (Ref.)
AG	50/408	2.38 (1.04–5.56)	2.36 (1.04–5.35)	360/664	2.18 (1.21–3.26)	2.13 (1.16–3.97)
AA	38/224	2.47 (1.11–5.47)	2.41 (1.01–5.78)	198/336	2.41 (1.34–3.84)	2.36 (1.28–3.96)
***HHEX_23* phenotype**						
G	102/856	0.80 (0.45–1.42)	0.78 (0.43–1.42)	694/1,334	0.70 (0.39–1.29)	0.69 (0.40–1.37)
A	126/856	2.43 (1.13–5.22)	2.28 (1.05–5.96)	756/1,336	2.22 (1.08–4.89)	2.04 (1.03–4.12)
A allele	982	1.35 (0.97–1.88)^c^		2,092	1.16 (0.97–1.39)^d^	
***IDE_9* genotype**						
TT	74/600	1.00 (Ref.)	1.00 (Ref.)	483/925	1.00 (Ref.)	1.00 (Ref.)
TC	37/235	1.07 (0.65–1.77)	1.08 (0.65–1.87)	214/372	1.23 (0.71–2.63)	1.18 (0.66–3.03)
CC	3/21	0.87 (0.66–2.56)	0.84 (0.56–2.66)	28/38	0.85 (0.77–3.11)	0.78 (0.70–3.21)
***IDE_9* phenotype**						
T	185/1,435	0.81 (0.46–1.44)	0.79 (0.463–1.43)	1,180/2,222	0.60 (0.53–1.65)	0.63 (0.44–1.54)
C	43/277	1.43 (0.93–5.22)	1.28 (0.85–4.96)	270/448	0.79 (0.66–3.29)	0.78 (0.71–4.02)
C allele	320	0.96 (0.89–1.94)		718	0.74 (0.69–1.38)	

^a^Number of participants with/without diabetes.

^b^Adjusted for age, sex, education, stroke, heart disease, SBP, DBP, BMI, and *APOE* ε4 status.

^c^ *p* = 0.066.

^d^ *p* = 0.065.

Over the 9-y follow-up of the KP (5,485 person-years; median per person, 5.5 y; range, 0.03–10.5 y), 358 participants were diagnosed with dementia, including 271 with AD. In fully adjusted Cox regression analysis, diabetes was associated with a more than 60% increased risk of dementia (HR 1.73, 95% CI 1.19–2.32) and AD (HR 1.66, 95% CI 1.06–2.40) after controlling for age, sex, education, baseline MMSE score, BMI, heart disease, stroke, SBP, DBP, *APOE* ε4, and survival status. Compared to *HHEX_23-*GG, the AG (HR 0.96, 95% CI 0.74–1.25) and AA (HR 1.05, 95% CI 0.78–1.40) genotypes were not significantly associated with incident dementia. We also found no significant association between *IDE_9*-TC (HR 1.04, 95% CI 0.83–1.31) or *IDE_9*-CC (HR 0.68, 95% CI 0.30–1.54) (*IDE_9*-TT as reference) and dementia.

In the SNAC-K, during the 6-y follow-up (8,371 person-years; median per person, 5.9 y; range, 2.07–7.60 y), 166 people developed dementia, including 121 with AD. Multi-adjusted Cox regression showed results similar to those of the KP regarding the association of diabetes, *HHEX_23*, and *IDE_9* with dementia and AD, adjusted for the confounders listed above. We found no significant association of *HHEX_23* and *IDE_9* genotypes with dementia and AD in either cohort (Table 3).

**Table 3:** Multi-adjusted hazard ratios and 95% CIs of dementia and Alzheimer disease related to diabetes, *HHEX_23*, *IDE_9*, and *APOE* in the Kungsholmen Project and SNAC-K.

Exposure/Genotype/Phenotype	Kungsholmen Project			SNAC-K		
	*n*	Dementia (*n* = 358)	AD (*n* = 271)	*n*	Dementia (*n* = 166)	AD (*n* = 121)
**Diabetes/prediabetes**						
No	856	1.00 (Ref.)	1.00 (Ref.)	1,335	1.00 (Ref.)	1.00 (Ref.)
Yes	114	1.73 (1.19–2.32)	1.66 (1.06–2.40)	725	1.68 (1.04–2.99)	1.64 (1.02–2.33)
***HHEX_23* genotype**						
GG	250	1.00 (Ref.)	1.00 (Ref.)	502	1.00 (Ref.)	1.00 (Ref.)
AG	458	0.96 (0.74–1.25)	1.02 (0.73–1.43)	1,024	0.89 (0.39–1.24)	1.04 (0.68–1.41)
AA	262	1.05 (0.78–1.40)	0.95 (0.70–1.28)	534	0.92 (0.35–1.36)	0.97 (0.74–1.37)
***HHEX_23* phenotype**						
G	708	1.02 (0.79–1.31)	1.01 (0.79–1.36)	1,526	0.91 (0.42–2.15)	0.93 (0.66–2.18)
A	720	1.01 (0.82–1.63)	0.98 (0.79–1.57)	1,558	1.01 (0.43–2.07)	1.00 (0.70–2.29)
A allele	982	1.41 (0.86–1.69)	1.39 (0.85–1.70)	2,092	1.52 (0.73–2.10)	1.47 (0.81–2.12)
***IDE_9* genotype**						
TT	674	1.00 (Ref.)	1.00 (Ref.)	1,408	1.00 (Ref.)	1.00 (Ref.)
TC	272	1.04 (0.83–1.31)	1.01 (0.79–1.28)	586	1.09 (0.87–1.39)	1.09 (0.88–1.41)
CC	24	0.68 (0.30–1.54)	0.69 (0.31–1.56)	66	0.47 (0.16–1.39)	0.46 (0.16–1.43)
***IDE_9* phenotype**						
T	976	1.01 (0.76–1.22)	1.00 (0.77–1.28)	1,994	0.93 (0.40–2.20)	0.96 (0.68–1.45)
C	296	0.93 (0.74–1.25)	0.95 (0.76–1.29)	652	0.98 (0.51–2.18)	1.00 (0.70–1.54)
C allele	320	1.00 (0.80–1.51)	0.96 (0.76–1.48)	718	0.95 (0.46–2.21)	1.01 (0.70–1.33)
***APOE* genotype**						
No ε4	706	1.00 (Ref.)	1.00 (Ref.)	1,487	1.00 (Ref.)	1.00 (Ref.)
Any ε4	264	1.34 (1.01–1.77)	1.42 (1.04–1.95)	573	1.47 (1.16–2.56)	1.56 (1.11–1.96)

Data are presented as HR (95% CI) and are multi-adjusted for age, sex, education, baseline MMSE score, BMI, heart disease, stroke, SBP, DBP, *APOE* ε4, and survival status. The results adjusted for age, sex, and education were similar to the multi-adjusted results (S5 Table).

### Joint Effect of *HHEX_23* and Diabetes on Dementia and Alzheimer Disease

The joint effect of diabetes and different genotypes of *HHEX_23* on the risk of dementia and AD was examined by creating dummy variables based on the joint exposures, and participants were divided into six groups. In the KP, multi-adjusted Cox regression analysis showed that the AA genotype in combination with diabetes was associated with a substantial risk of dementia (HR 5.54, 95% CI 2.40–7.18) and AD (HR 4.81, 95% CI 1.88–8.50) compared to the GG genotype without diabetes after controlling for age, sex, education, baseline MMSE score, BMI, heart disease, stroke, SBP, DBP, *APOE* ε4, and follow-up survival status. In the SNAC-K, the multi-adjusted HRs for dementia (4.87, 95% CI 1.91–5.66) and AD (4.01, 95% CI 1.72–6.37) were much greater among the *HHEX–23-*AA carriers with diabetes than among the *HHEX_23-*GG carriers without diabetes (Table 4).

**Table 4:** Hazard ratios and 95% CIs of dementia and Alzheimer disease related to diabetes (including prediabetes) by *HHEX–23* genotype in the Kungsholmen Project and SNAC–K.

Joint Exposure		Kungsholmen Project					SNAC-K				
Diabetes	*HHEX-23*	*n*	Dementia (*n* = 358)		AD (*n* = 271)		*n*	Dementia (*n* = 166)		AD (*n* = 121)	
			*n*	HR (95% CI)^a^	*n*	HR (95% CI) ^a^		*n*	HR (95% CI) ^a^	*n*	HR (95% CI) ^a^
No	GG	224	88	1.00 (Ref.)	71	1.00 (Ref.)	335	26	1.00 (Ref.)	19	1.00 (Ref.)
Yes	GG	26	11	0.88 (0.38–2.03)	7	0.95 (0.38–2.39)	167	18	0.71 (0.40–2.46)	12	0.79 (0.34–2.57)
No	AG	408	140	0.90 (0.70–1.17)	107	0.95 (0.71–1.28)	664	52	0.87 (0.46–1.21)	43	0.82 (0.41–1.50)
Yes	AG	50	13	2.27 (0.88–9.45)	9	2.76 (0.90–9.70)	360	29	1.79 (0.72–4.53)	20	1.66 (0.61–4.61)
No	AA	224	95	0.99 (0.74–1.32)	69	1.01 (0.72–1.41)	336	24	0.77 (0.47–1.16)	16	0.72 (0.40–1.20)
Yes	AA	38	11	5.54 (2.40–7.18)	8	4.81 (1.88–8.50)	198	17	4.78 (1.91–5.66)	11	4.01 (1.72–6.37)

^a^Adjusted for age, sex, education, baseline MMSE score, follow-up survival status, BMI, heart disease, stroke, SBP, DBP, and *APOE* ε4. The results adjusted for age, sex, and education were similar to the multi-adjusted results (S6 Table).

In the KP, the HR for the statistical interaction between *HHEX_23*-AA and diabetes was 4.79 (95% CI 1.63–8.90, *p* = 0.013) for dementia and 3.55 (95% CI 1.45–9.91, *p* = 0.025) for AD after adjustment for age, sex, education, baseline MMSE score, follow-up survival status, BMI, heart disease, stroke, SBP, DBP, and *APOE* ε4. In SNAC-K, the multi-adjusted HR for the interaction was 4.06 (95% CI 1.06–7.58, *p* = 0.039) for dementia and 3.29 (95% CI 1.02–8.33, *p* = 0.044) for AD. These results suggest that *HHEX_23* may interact with diabetes to be greatly associated with the risk of dementia and AD.

In pooled data analysis, including data from both the KP and the SNAC-K, the adjusted HR of *HHEX_23*-AA in combination with diabetes was 5.01 (95% CI 1.93–5.86) for dementia and 4.46 (95% CI 1.79–5.28) for AD compared to the GG genotype without diabetes. The multi-adjusted HR for the interaction between the AA genotype and diabetes on dementia and AD was 4.01 (95% CI 1.24–6.67, *p* = 0.009) for dementia and 3.32 (95% CI 1.21–7.26, *p* = 0.012) for AD.

There were no statistically significant interactions between the *HHEX_23*-AA genotype and *APOE* ε4 on dementia. In the KP, the HR for the interaction was 1.40 (95% CI 0.82–2.41) for dementia and 1.64 (95% CI 0.88–3.20) for AD after adjustment for age, sex, education, baseline MMSE score, follow-up survival status, BMI, heart disease, stroke, SBP, and DBP. Similar results on the interactions were also shown from the SNAC-K. We found no joint effects of *IDE_9* and diabetes on dementia and AD (S1 Table).

**Fig 1:**
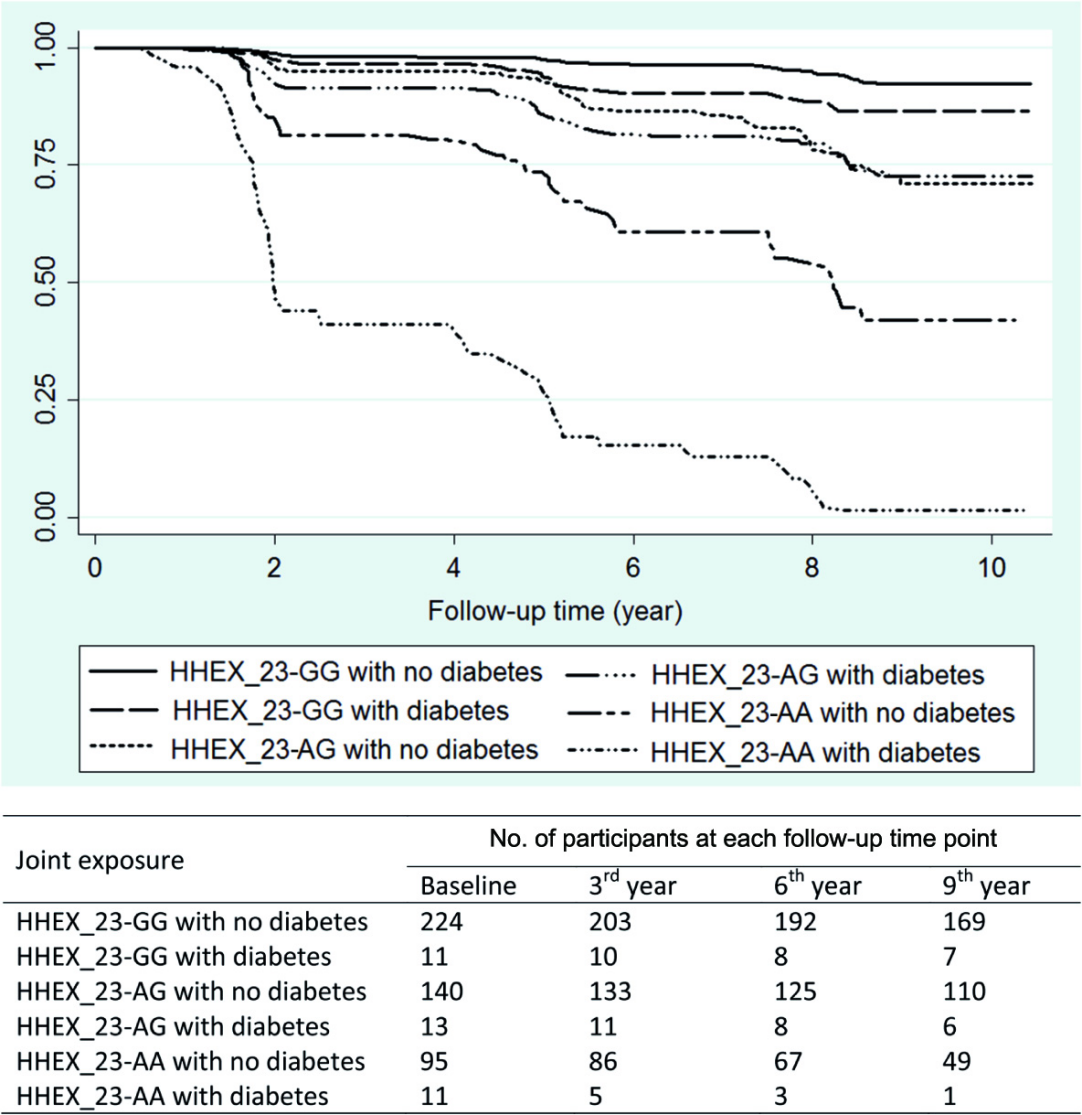
Kaplan–Meier survival estimates from baseline to dementia occurrence by diabetes (including prediabetes) and *HHEX_23* genotype (adjusted for age, sex, and education).

Kaplan–Meier survival analysis showed that the median time from baseline to dementia occurrence in the KP was 1.83 y (95% CI 1.44–4.24) among *HHEX_23*-AA carriers with diabetes and 5.01 y (95% CI 4.15–7.92) in *HHEX_23-*GG carriers without diabetes. Thus, *HHEX_23*-AA and diabetes accelerated dementia onset by an average of 3.18 y (Fig 1).

### *HHEX_23*-AA and Diabetes in Relation to Structural Brain Changes

**Fig 2:**
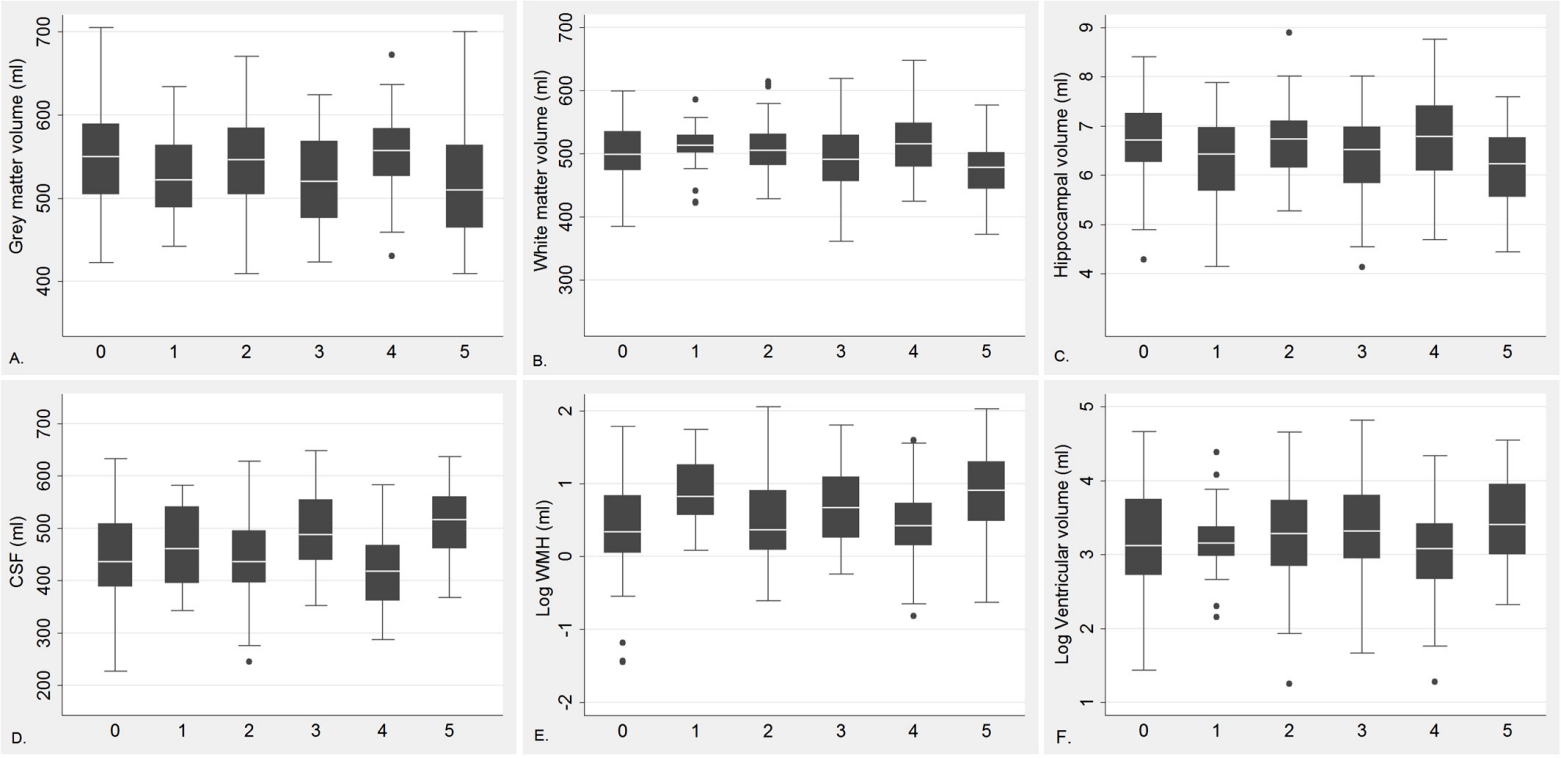
Regional brain volume by diabetes (including prediabetes) and *HHEX_23* genotype among dementia-free individuals. 0 = GG genotype with no diabetes; 1 = GG genotype with diabetes; 2 = AG genotype with no diabetes; 3 = AG genotype with diabetes; 4 = AA genotype with no diabetes, and 5 = AA genotype with diabetes. GMV (A), WMV (B), hippocampal volume (C), CSF volume (D), WMH (WMH/total tissue volume × 1,000) (E), and log transformed lateral ventricular volume (F). These regions are adjusted for ICV.

The characteristics of the MRI study population were generally similar, except education and diabetes (S2 Table), among participants with different *HHEX_23* genotypes. Participants carrying *HHEX_23*-AA with diabetes had smaller adjusted GMV, WMV, and hippocampal volume, but greater CSF volume, WMH, and lateral ventricular volume, than those without these conditions (Fig 2). In linear regression analysis, *HHEX_23*-AA in combination with diabetes was significantly related to lower GMV (β = −34.25, *p* = 0.014), WMV (β = −33.24, *p* = 0.001), and hippocampal volume (β = −0.51, *p* = 0.003), but greater CSF volume (β = 67.49, *p* < 0.001), WMH (β = 9.46, *p* = 0.002), and lateral ventricular volume (β = 0.31, *p* = 0.012) compared to the GG genotype without diabetes (Table 5). The interaction between the AA genotype and diabetes on WMV (β = −22.80, 95% CI −37.11 to −8.49, *p* = 0.008) and CSF volume (β = 23.85, 95% CI 4.85–42.85, *p* = 0.014) remained statistically significant. These structural neuroimaging data on dementia-free people provide further support for our observational findings. We found no statistically significant association between these brain structural changes and diabetes by *IDE_9* (S3 Table).

**Table 5:** Linear regression β coefficients and 95% CIs of adjusted brain characteristics in relation to diabetes (including prediabetes) by *HHEX_23* genotype (*n* = 338).

Joint Exposure		Number of Participants	GMV^a^		WMV^a^		Hippocampal Volume^a^		CSF Volume^a^		WMH/Total TissueVolume		Lateral Ventricular Volume^a^	
Diabetes	*HHEX_23*		β (95% CI)	*p-*Value	β (95% CI)	*p-*Value	β (95% CI)	*p-*Value	β (95% CI)	*p-*Value	β (95% CI)	*p-*Value	β (95% CI)	*p-*Value
No	GG	61	Ref.		Ref.		Ref.		Ref.		Ref.		Ref.	
Yes	GG	20	−22.81 (−51.10 to 5.47)	0.113	6.08 (−20.24 to 32.41)	0.650	−0.46 (−0.88 to 0.16)	0.109	16.72 (−22.79 to 56.26)	0.406	6.94 (−0.95 to 14.84)	0.085	0.14 (−0.16 to −0.45)	0.356
No	AG	98	−5.13 (−22.3 to 11.99)	0.556	4.28 (−11.66 to 20.23)	0.597	−0.03 (−0.29 to 0.22)	0.813	0.85 (−23.09 to 24.79)	0.944	0.67 (−4.01 to 5.36)	0.777	0.91 (−0.10 to 0.28)	0.348
Yes	AG	58	−29.56 (−48.95 to −10.17)	0.003	−12.82 (−30.88 to 5.22)	0.163	−0.30 (−0.59 to −0.01)	0.048	42.39 (15.28 to 69.49)	0.003	2.76 (−2.57 to 8.08)	0.309	0.20 (−0.02 to 0.41)	0.069
No	AA	62	5.78 (−13.31 to 24.87)	0.502	13.92 (−3.85 to 31.69)	0.124	0.06 (−0.22 to 0.34)	0.694	−19.70 (−46.39 to 6.98)	0.147	−2.00 (−7.23 to 3.22)	0.452	−0.14 (−0.35 to 0.06)	0.165
Yes	AA	39	−34.25 (−56.07 to −12.44)	0.014	−33.24 (−53.54 to −12.93)	0.001	−0.51 (−0.83 to −0.16)	0.003	67.49 (37.01 to 97.97)	0.000	9.46 (3.39 to 15.52)	0.002	0.31 (0.07 to 0.55)	0.012

^a^These regions are adjusted for ICV, age, sex, and education.

Compared to the remaining SNAC-K sample (*n* = 1,722), the MRI sample was younger (mean ± standard deviation: 71.1 ± 9.1 versus 75.5 ± 11.4 y, *p* < 0.001), included more men (41.4% versus 35.0%, *p* = 0.003), and received more years of education (mean ± standard deviation: 12.6 ± 4.1 versus 11.7 ± 4.0 y, *p* < 0.001).

### Supplementary Analyses

Similar results were obtained when Cox regression analyses were repeated using age as the time scale, when diabetes and prediabetes were treated as separate exposures, when diabetes was treated as a time-dependent variable considering incident diabetes at follow-up time points, and when the analyses were repeated among participants who survived until the time when dementia status was determined (*n* = 170, including 136 with dementia and 115 with AD). As diabetes was significantly associated with an elevated mortality (HR 1.48, 95% CI 1.06–2.06), the analysis was repeated using competing risk regression where competing death was taken into account, which produced results that were very similar to those from the initial analysis. A sensitivity analysis using multiple imputed data showed results similar to those from the initial analysis. As the KP population was aged ≥75 y, we repeated the analysis among only the participants aged ≥75 y in the SNAC-K, which produced results that were similar to those from the whole population. We also repeated the analysis on the *IDE_9*–diabetes association after excluding prediabetes, and the association remained non-significant (S4 Table). Finally, similar analyses for the role of rs2251101 (*IDE_7*), rs12783634, and rs1999763 in the diabetes–dementia association were performed, and no statistically significant results were obtained.

## Discussion

In this population-based study consisting of two cohorts of older adults and a subsample with MRI, we found that the *HHEX_23*-AA genotype interacts with diabetes to substantially increase the risk of dementia and AD by more than four times. Further, we found that people with diabetes carrying the AA genotype had significantly smaller hippocampal and gray and white matter volumes, along with greater lateral ventricular volume and WMH, than those without these conditions. We found no joint effect of *IDE_9* and diabetes on dementia and AD. Our findings suggest that a genetic variant in *HHEX_23* may play an important role in the development of dementia and AD among people with diabetes.

The main strengths of our study are the population-based cohort, the long-term prospective study design, and the assessment of diabetes at baseline and each follow-up examination. In addition, the replication cohort population was located in the same area as the initial cohort, and had MRI data available. However, some limitations should be noted. First, we used random blood glucose to define diabetes and prediabetes at baseline. As the receiver operating curve is 0.75 (0.49–0.80) for detecting undiagnosed diabetes using random blood glucose [16], impaired glucose tolerance might have been misclassified as non-diabetes and non-prediabetes, which would bias the results of our overall and subgroup analyses toward the null hypothesis. Second, we were unable to assess whether the joint effect of diabetes and *IDE/HHEX* genes on dementia is mediated by insulin resistance, because data on plasma insulin levels were not available in our study. Third, diabetes and dementia are both associated with elevated mortality [16], which may lead to an underestimation of the strength of the diabetes–dementia or diabetes–AD association because of selective survival. However, similar results were obtained when we used competing risk regression to analyze the association. Fourth, compared to genome-wide association studies, the number of genes examined in this study was very small. However, these genes were selected based on previous studies using the Swedish population. Focusing on the targeted genes allowed us to have more possibilities and spaces to systematically verify our hypothesis in two cohorts and an MRI study. Fifth, both the KP and the SNAC-K are prospective studies of community-based cohorts that included older adults living in a geographically defined central area in Stockholm. The dropout rate in the screening phase was 23.6% in the KP and 26.7% in the SNAC-K, mainly because of death, refusal, or moving from the area [21,23]. In fact, after excluding those who died, the rate of participation in each phase was quite high, varying between 85% and 94%. Compared to those who participated in the screening phase, the dropouts due to death were older and more likely to be men. However, individuals who refused to participate or who moved did not differ from participants in terms of major demographic features [39,40]. A sensitivity analysis using imputed data showed results similar to those from the initial analysis. Thus, we believe the two study populations could be representative of the local general populations at the time the studies started. As the allelic frequencies of the genes examined did not differ from their expected frequencies and were comparable to those in other populations, the major findings from this study may be generalized to populations aged ≥60 y in Western societies with characteristics similar to those in the Kungsholmen population. However, caution is needed when generalizing our findings to younger or rural populations. Finally, it could be considered a limitation that the diagnoses of dementia were made on a clinical basis. However, the clinical assessment for dementia was comprehensive and validated [31]. Moreover, the results from the MRI data in this study provided further support for our findings even among dementia-free individuals.

IDE is a large zinc-binding protease of the M16A metalloprotease subfamily known to cleave multiple short polypeptides. IDE is ubiquitously expressed, with its highest expression in the liver, testes, muscle, and brain [7]. *HHEX_23* is a protein-coding gene. This gene encodes a member of the homeobox family of transcription factors, many of which are involved in developmental processes. DNA polymorphisms in *IDE/HHEX* genes contribute to variation in plasma insulin levels, which is important for the maintenance of insulin concentrations [41]. Variations in *HHEX*_23 may affect *IDE* expression through a primary effect upon *IDE* mRNA levels [10]. It has been suggested that the expression of *IDE* can be affected by aging, and its activity is significantly decreased with age [42]. However, the effect of the *HHEX_23* alleles on *IDE* expression is unclear.

In a recent meta-analysis including 11 studies comprising 5,771 cases and controls, a non-significant association between *HHEX_23* and AD was shown [43]. In the current study, we did not find a significant association between *HHEX_23* genotype and the development of dementia and AD among all participants, but we did find an association among people with diabetes. These results may explain the discrepancies in the association between *HHEX_23* and AD in previous studies that did not take diabetes into account.

*IDE* genes have shown suggestive linkage with type 2 diabetes in a number of linkage studies [12], and the *IDE/HHEX* genomic region has been associated with type 2 diabetes in genome-wide association studies [44–46]. The relation of *HHEX_23* and *IDE_9* with type 2 diabetes has been reported in several studies [13,47,48], but there are some negative findings [44,49,50]. A study of Swedish men showed a significant association of *HHEX_23*, but not *IDE_9*, with diabetes [51]. A large-scale gene-centric meta-analysis across 39 studies for identification of type 2 diabetes loci also failed to identify rs1887922 as being associated with diabetes [52]. In our study, we observed that, compared with the *HHEX_23*-GG genotype, the AA genotype was associated with diabetes and prediabetes, but we did not find a significant association between *IDE_9* and diabetes. Possible explanations for the mixed findings between studies may be age differences between study populations. The age of our study population is much older than that in other studies (mean age of 78 versus 35–65 y) [13,47–50]. As diabetes is associated with mortality, the proportion of individuals with a severe form of diabetes (this could be more related to *IDE_9*) might be less than that in other studies because of selective survival. Another explanation could be differences in study design and diabetes assessment (such as inclusion of prediabetes). We repeated the analysis after excluding prediabetes, and the *IDE_9–*diabetes association remained non-significant (S4 Table). Thus, further research is needed to clarify the association between *IDE_9* and diabetes.

As IDE demonstrates an ability to degrade insulin, amylin, and Aβ, it has been suggested that *IDE* is a candidate gene for both type 2 diabetes and AD [7]. Thus, this gene may explain risk for both AD and diabetes, and perhaps even the reciprocal risk between the two disorders. Animal studies have revealed that dysfunctional IDE protease causes diabetic and neuro-pathologic changes in Goto-Kakizaki rats [53,54]. However, no studies to our knowledge have examined the possible relationship between the variations of coding sequences of the *IDE/HHEX* genes and the two diseases, type 2 diabetes and AD, in humans. In the present study, we found that the *HHEX_23*-AA genotype may substantially increase the risk of dementia and AD among elderly individuals with diabetes. Our findings suggest that diabetes-related AD might occur mainly among *HHEX_23*-AA carriers.

To further support our observations, MRI data showed that among dementia-free people, *HHEX_23*-AA together with diabetes was associated with significantly lower regional brain volumes and greater WMH, which are the early brain changes related to dementia and AD before disease onset, compared to non-diabetic participants with the GG genotype. We found that diabetes interacts with the AA genotype to be related to more reduction of WMV and increase of CSF volume. The explanation for our main findings could be that not only may dementia and diabetes compound each other’s pathologies, but they may interact on the same molecular pathways [55]. It would be very interesting to carry out experiments based on AD tissue expression data to further confirm our findings. However, there are some challenges in assessing *IDE* gene expression. First, the molecular basis of functional polymorphism in *IDE* remains incompletely resolved, though limited data have shown that variable mRNA expression is important [10]. Second, deciphering the character of functional polymorphism in the *IDE* region is a daunting task when allelic heterogeneity is present. Third, RNA-seq studies produce large and complex datasets, and data analysis is further challenged by technical issues. Finally, protocol-specific bias may under- or overrepresent specific loci, leading to biased results; thus, careful data quality control and normalization are necessary [56].

In summary, our study provides the first evidence, to our knowledge, that *IDE*/*HHEX* genes—including polymorphisms in *HHEX_23*, but not *IDE_9*—play an important role in the association between diabetes, dementia, and structural brain changes. Our results support the hypothesis that variations within the *IDE/HHEX* gene region may underlie such an association. Our findings highlight the need to control diabetes in order to prevent dementia and AD, especially among people carrying *HHEX_23*-AA. Further epidemiological studies and experiments based on AD tissue expression data are warranted to clarify the role of *HHEX_23* in the diabetes–dementia association and the mechanisms behind the given association.

## Supporting Information

S1 Checklist STROBE checklist. (DOCX)

S1 Table Hazard ratios and odds ratios and 95% CIs for dementia and Alzheimer disease related to diabetes (including prediabetes) by *IDE_9* genotype in the Kungsholmen Project (*n* = 970) and the SNAC-K (*n* = 2,060). (DOCX)

S2 Table Characteristics of the dementia-free participants in the SNAC-K MRI study population by *HHEX_23* genotype (*n* = 338). (DOCX)

S3 Table Linear regression β coefficients and 95% CIs for adjusted brain characteristics in relation to diabetes (including prediabetes) by *IDE_9* genotype (*n* = 338). HV, hippocampal volume; LVV, lateral ventricular volume; TTV, total tissue volume.
(DOCX)

S4 Table Crude and multi-adjusted odds ratios and 95% CIs for the relation of *IDE_9* to diabetes (excluding prediabetes) in the Kungsholmen Project and the SNAC-K. (DOCX)

S5 Table Basic adjusted hazard ratio and 95% CI for dementia and Alzheimer disease related to diabetes, *HHEX_23*, *IDE_9*, and *APOE* in the Kungsholmen Project and the SNAC-K. (DOCX)

S6 Table Basic adjusted hazard ratios with 95% CIs for dementia and Alzheimer disease related to diabetes (including prediabetes) by *HHEX-23* genotype in the Kungsholmen Project and the SNAC-K. (DOCX)

## Abbreviations:

AD: Alzheimer disease
Aβ: β-amyloid
BMI: body mass index
CSF: cerebral spinal fluid
DBP: diastolic blood pressure
*DSM-III-R*: *Diagnostic and Statistical Manual of Mental Disorders*, *Third Edition–Revised*
FLAIR: fluid-attenuated inversion recovery
GMV: gray matter volume
HR: hazard ratio
ICV: intracranial volume
MMSE: Mini-Mental State Examination
KP: Kungsholmen Project
OR: odds ratio
SBP: systolic blood pressure
SNAC-K: Swedish National Study on Aging and Care–Kungsholmen
WMH: white matter hyperintensity
WMV: white matter volume
