# Supplementary material for: HHEX_23 AA Genotype Exacerbates Effect of Diabetes on Dementia and Alzheimer Disease: A Population-Based Longitudinal Study
Source: PLoS Med. 2015 Jul 14;12(7):e1001853. doi: 10.1371/journal.pmed.1001853 (PMC4501827; doi:10.1371/journal.pmed.1001853)
Supplement: S1 Checklist — (DOCX) [file pmed.1001853.s001.docx]

STROBE Statement—checklist of items that should be included in reports of observational studies (MS ID#: PMEDICINE-D-14-02434R1 by Xu et al.)

|  | Item No. | Recommendation | Page  No. | Relevant text from manuscript |
| --- | --- | --- | --- | --- |
| **Title and abstract** | 1 | (*a*) Indicate the study’s design with a commonly used term in the title or the abstract | 1 | A population-based longitudinal study |
|  |  | (*b*) Provide in the abstract an informative and balanced summary of what was done and what was found | 2 | Explored the role of *IDE/HHEX* genes in the diabetes-dementia association. We found that the *HHEX_23* gene interacts with diabetes to increase dementia risk. |
| Introduction | | | |  |
| Background/rationale | 2 | Explain the scientific background and rationale for the investigation being reported | 3 | Expression of *IDE* influences the rate of insulin (related to diabetes) and Aβ degradation (marker of Alzheimer’s disease, AD). Thus, we hypothesize that *IDE/HHEX* genes may play a role in the diabetes-dementia association. |
| Objectives | 3 | State specific objectives, including any prespecified hypotheses | 4 | To explore whether variations in these genes within *IDE/HHEX* region play a role in the association of diabetes with dementia and AD |
| Methods | | | |  |
| Study design | 4 | Present key elements of study design early in the paper | 4 | This is a population-based prospective study on aging and dementia |
| Setting | 5 | Describe the setting, locations, and relevant dates, including periods of recruitment, exposure, follow-up, and data collection | 4-7 | A community of Kungsholmen, Stockholm Recruitment: 1987 1989.  Exposure: Diabetes and prediabetes, *HHEX_23* (rs1544210), and *IDE_9* (rs1887922)  Follow-up: 9 years  Data collection: made at baseline and follow-ups |
| Participants | 6 | (*a*) *Cohort study*—Give the eligibility criteria, and the sources and methods of selection of participants. Describe methods of follow-up  *Case-control study*—Give the eligibility criteria, and the sources and methods of case ascertainment and control selection. Give the rationale for the choice of cases and controls  *Cross-sectional study*—Give the eligibility criteria, and the sources and methods of selection of participants | 4-5  6 | All inhabitants living in the area and aged 75+ on 1 Oct. 1987. During a 9-year follow-up, three clinical examinations were carried out at an average interval of 3 years till 1997-1998.  The MRI sample was taken from non-disabled and non-demented participants of SNAC-K during 2001-2003, and 552 consecutive subjects received structural MRI |
|  |  | (*b*) *Cohort study*—For matched studies, give matching criteria and number of exposed and unexposed  *Case-control study*—For matched studies, give matching criteria and the number of controls per case |  |  |
| Variables | 7 | Clearly define all outcomes, exposures, predictors, potential confounders, and effect modifiers. Give diagnostic criteria, if applicable | 6-8 | Data collection, assessment of diabetes and prediabetes, SNP genotyping, dementia and AD diagnosis and MRI reading protocol |
| Data sources/ measurement | 8* | For each variable of interest, give sources of data and details of methods of assessment (measurement). Describe comparability of assessment methods if there is more than one group | 6-8 | Diabetes and prediabetes: WHO (1999)  SNP: Sequenom MSAA Array^TM^ platform  Dementia and AD: DSM-III-R & NINCDS-ADRDA criteria. |
| Bias | 9 | Describe any efforts to address potential sources of bias | 9-11, 14 | Comparisons between participants and dropouts, sensitivity analysis and multiple imputation. |
| Study size | 10 | Explain how the study size was arrived at | 5-6 | After exclusion dropouts and missing values, 970 in KP, and 2060 in SNAC-K were left. |

Continued on next page

| Quantitative variables | 11 | Explain how quantitative variables were handled in the analyses. If applicable, describe which groupings were chosen and why | 6-8 | Education, BMI, blood pressure, blood glucose, and regional brain volumes on MRI. |
| --- | --- | --- | --- | --- |
| Statistical methods | 12 | (*a*) Describe all statistical methods, including those used to control for confounding | 9 | Cox, logistic and linear regressions controlling for a number of potential confounders. |
|  |  | (*b*) Describe any methods used to examine subgroups and interactions | 9 | Interaction terms were used. |
|  |  | (*c*) Explain how missing data were addressed | 9 | Multiple imputation and exclusions |
|  |  | (*d*) *Cohort study*—If applicable, explain how loss to follow-up was addressed  *Case-control study*—If applicable, explain how matching of cases and controls was addressed  *Cross-sectional study*—If applicable, describe analytical methods taking account of sampling strategy | 11, 14 | By comparisons between participants and dropouts, inclusions through multiple imputations. For died subjects, competing risk was taken into account. |
|  |  | (*e*) Describe any sensitivity analyses | 9 | Used only survivals, and leaving out or imputing all missing values. |
| Results | | | | |
| Participants | 13* | (a) Report numbers of individuals at each stage of study—eg numbers potentially eligible, examined for eligibility, confirmed eligible, included in the study, completing follow-up, and analysed | 4-6 | Of 1700 eligible, 1475 dementia-free were examined at baseline, and 970 left after exclusions of subjects with dementia and missing blood samples, and refusals. |
|  |  | (b) Give reasons for non-participation at each stage | 5 | During follow-up, 346 died and 51 dropped out |
|  |  | (c) Consider use of a flow diagram |  | Published in previous study cited in the MS (Xu WL et al, 2009). |
| Descriptive data | 14* | (a) Give characteristics of study participants (eg demographic, clinical, social) and information on exposures and potential confounders | 10, 24 | As shown in Table 1 and text. |
|  |  | (b) Indicate number of participants with missing data for each variable of interest |  | As participants with missing values were excluded, no missing was present for subjects included in this study. |
|  |  | (c) *Cohort study*—Summarise follow-up time (eg, average and total amount) | 11 | 9 year (5485 person years, median 5.5 years, range 0.03-10.5) |
| Outcome data | 15* | *Cohort study*—Report numbers of outcome events or summary measures over time | 11 | 358 dementia including 271 AD |
|  |  | *Case-control study—*Report numbers in each exposure category, or summary measures of exposure |  |  |
|  |  | *Cross-sectional study—*Report numbers of outcome events or summary measures | 13, Fig 1 | Regional brain volumes in each category |
| Main results | 16 | (*a*) Give unadjusted estimates and, if applicable, confounder-adjusted estimates and their precision (eg, 95% confidence interval). Make clear which confounders were adjusted for and why they were included | 25-26 | Basic- and multi-adjusted estimates were shown in tables |
|  |  | (*b*) Report category boundaries when continuous variables were categorized |  |  |
|  |  | (*c*) If relevant, consider translating estimates of relative risk into absolute risk for a meaningful time period | 11-12, 14-15 | Diabetes increased dementia risk by 60%, but greatly higher when combined with *HHEX_23*-AA genotype.  *HHEX_23-AA* genotype interacts with diabetes to substantially increase the risk of dementia and AD by more than three times. |

Continued on next page

| Other analyses | 17 | Report other analyses done—eg analyses of subgroups and interactions, and sensitivity analyses | 14 | Sensitivity analysis, multiple imputation and Competing risk regression analysis were performed. | |
| --- | --- | --- | --- | --- | --- |
| Discussion | | | | |  |
| Key results | 18 | Summarise key results with reference to study objectives | 14 | *HHEX_23*-AA genotype may interact with diabetes to greatly increase the risk of dementia and AD. |  |
| Limitations | 19 | Discuss limitations of the study, taking into account sources of potential bias or imprecision. Discuss both direction and magnitude of any potential bias | 15-16 | Random blood glucose was used and no insulin measure was available. *IDE* genes were selected not cover all SNPs comparing to GWAS. The strength of the given association might have been underestimated. |  |
| Interpretation | 20 | Give a cautious overall interpretation of results considering objectives, limitations, multiplicity of analyses, results from similar studies, and other relevant evidence | 15-16 | Given association could be underestimated, and may not be applicable in young population. |  |
| Generalisability | 21 | Discuss the generalisability (external validity) of the study results | 16 | May be generalized to the urban population aged 60+ in Western society. |  |
| Other information | |  | | |  |
| Funding | 22 | Give the source of funding and the role of the funders for the present study and, if applicable, for the original study on which the present article is based | 19 | Ministry of health and Social Affairs, Swedish Research Council, the board of Research at KI, and private foundations. |  |

*Give information separately for cases and controls in case-control studies and, if applicable, for exposed and unexposed groups in cohort and cross-sectional studies.

**Note:** An Explanation and Elaboration article discusses each checklist item and gives methodological background and published examples of transparent reporting. The STROBE checklist is best used in conjunction with this article (freely available on the Web sites of PLoS Medicine at http://www.plosmedicine.org/, Annals of Internal Medicine at http://www.annals.org/, and Epidemiology at http://www.epidem.com/). Information on the STROBE Initiative is available at www.strobe-statement.org.
