# Supplementary material for: HHEX_23 AA Genotype Exacerbates Effect of Diabetes on Dementia and Alzheimer Disease: A Population-Based Longitudinal Study
Source: PLoS Med. 2015 Jul 14;12(7):e1001853. doi: 10.1371/journal.pmed.1001853 (PMC4501827; doi:10.1371/journal.pmed.1001853)
Supplement: S1 Table — (DOCX) [file pmed.1001853.s002.docx]

**S1 Table. Hazard ratios (HRs) and odds ratios (ORs) with confidence intervals (CIs) of dementia and Alzheimer’s disease related to diabetes (including prediabetes) by *IDE_9* genotypes in the Kungsholmen Project (*n* = 970) and SNAC-K study (*n* = 2,060).**

| **Joint exposure** | |  | **The Kungsholmen project** | | |  | **The SNAC-K study** | | | |
| --- | --- | --- | --- | --- | --- | --- | --- | --- | --- | --- |
| Diabetes | *IDE_9* |  | *n* | **Dementia** (*n* = 358) | **AD** (*n* = 271) |  | | *n* | **Dementia** (*n* = 166) | **AD** (*n* = 121) |
|  |  |  |  | *n*  HR (95% CI)^a^ | *n*  HR (95% CI)^a^ |  |  |  | *n*  HR (95% CI)^a^ | *n*  HR (95% CI)^a^ |
| No | TT |  | 600 | 221 1.00 (Ref.) | 168 1.00 (Ref.) |  | | 925 | 83 1.00 (Ref.) | 68 1.00 (Ref.) |
| Yes | TT |  | 74 | 26 1.37 (0.87–2.81) | 17 1.63 (0.82–2.79) |  | | 483 | 35 1.12 (0.83–1.62) | 25 1.06 (0.74–1.88) |
| No | TC |  | 235 | 96 1.11 (0.87–1.41) | 76 1.03 (0.80–1.31) |  | | 372 | 24 0.94 (0.77–1.46) | 15 0.78 (0.62–1.50) |
| Yes | TC |  | 37 | 7 1.12 (0.43–1.95) | 6 1.46 (0.68–3.12) |  | | 214 | 17 0.98 (0.70–1.62) | 9 0.67 (0.64–1.752 |
| No | CC |  | 21 | 6 0.72 (0.32–1.62) | 3 0.70 (0.31–1.58) |  | | 38 | 4 1.08 (0.62–3.61) | 2 0.98 (0.47–3.79) |
| Yes | CC |  | 3 | 2 2.11 (0.01–9.98) | 1 2.10 (0.01–10.50) |  | | 28 | 3 2.24 (0.79–3.97) | 2 2.06 (0.65–4.47) |

^a^ Adjusted for age, sex, education, baseline MMSE score, follow–up survival status, body mass index, heart disease, stroke, systolic blood pressure, diastolic blood pressure, and *APOE* ε4.
