# Supplementary material for: HHEX_23 AA Genotype Exacerbates Effect of Diabetes on Dementia and Alzheimer Disease: A Population-Based Longitudinal Study
Source: PLoS Med. 2015 Jul 14;12(7):e1001853. doi: 10.1371/journal.pmed.1001853 (PMC4501827; doi:10.1371/journal.pmed.1001853)
Supplement: S2 Table — (DOCX) [file pmed.1001853.s003.docx]

**S2 Table. Characteristics of the dementia–free participants in the SNAC–K MRI study population by *HHEX_23* genotype (*n* = 338).**

| **Characteristics** | **GG** | **AG** | **AA** | *p-value* |
| --- | --- | --- | --- | --- |
| Participants | 81 | 156 | 101 | 0.176^b^ |
| Age (years) | 72.7 (9.8) | 74.3 (9.1) | 73.3 (9.2) | 0.436 |
| Female sex | 46 (56.8) | 95 (60.9) | 57 (56.4) | 0.725 |
| Educational level ≥8 years | 67 (82.7) | 121 (77.6) | 94 (93.1) | 0.005 |
| MMSE score | 28.8 (1.5) | 28.9 (1.2) | 29.0 (1.4) | 0.622 |
| Diabetes/Pre–diabetes | 15 (18.5) | 49 (31.4) | 33 (33.3) | 0.040 |
| Body mass index (kg/m^2^) | 26.2 (2.7) | 25.7 (4.0) | 25.4 (3.4) | 0.277 |
| Any *APOE* ε4 allele | 26 (32.1) | 33 (21.3) | 31 (31.0) | 0.108 |
| Grey matter volume (ml) | 543.4 (82.6) | 530.8 (70.6) | 542.9 (69.3) | 0.304 |
| White matter volume (ml) | 501.7 (76.5) | 493.2 (72.2) | 500.9 (81.1) | 0.624 |
| Hippocampal volume (ml) | 7.4 (1.1) | 7.2 (0.9) | 7.3 (1.1) | 0.433 |
| Cerebrospinal fluid (ml) | 445 (99.3) | 453 (92.5) | 459.6 (105.8) | 0.616 |
| Lateral ventricular volume^a^ (ml) | 22.7 | 27.1 | 25.5 | 0.215 |
| White matter hyperintensity^a^ (ml) | 2.8 | 3.0 | 3.1 | 0.751 |
| Intracranial volume (ml) | 1490.2 (176.2) | 1477.2 (151.7) | 1503.5 (160.2) | 0.436 |

Figures are mean (SD), or numbers (percentages) of participants.

^a^ Values are medians.

^b^ *p-values* for Hardy–Weinberg equilibrium.
