# Supplementary material for: HHEX_23 AA Genotype Exacerbates Effect of Diabetes on Dementia and Alzheimer Disease: A Population-Based Longitudinal Study
Source: PLoS Med. 2015 Jul 14;12(7):e1001853. doi: 10.1371/journal.pmed.1001853 (PMC4501827; doi:10.1371/journal.pmed.1001853)
Supplement: S3 Table — HV, hippocampal volume; LVV, lateral ventricular volume; TTV, total tissue volume. (DOCX) [file pmed.1001853.s004.docx]

**S3 Table.** **Linear regression β coefficients and 95% confidence intervals (CIs) of adjusted grey matter volume (GMV), white matter volume (WMV), hippocampal volume (HV), cerebralspinal fluid (CSF), and white matter hyperintensity/total tissue volume (WMH/TTV) and lateral and ventricular volume (LVV) in relation to diabetes (including prediabetes) by *IDE_9* genotypes (*n* = 338).**

| **Joint exposure** | | **GMV**^a^ | *p-value* | **WMV**^a^ | *p-value* | **HV**^a^ | *p-value* |
| --- | --- | --- | --- | --- | --- | --- | --- |
| Diabetes *IDE_9* | | β (95% CI) |  | β (95% CI) |  | β (95% CI) |  |
| No | TT | Ref. |  | Ref. |  | Ref. | |
| Yes | TT | -10.57 (-29.05 – 7.89) | 0.260 | -7.02 (-26.11 – 12.06) | 0.468 | -0.10 (-0.35 – 0.16) | 0.458 |
| No | TC | -7.75 (-30.04 – 14.52) | 0.493 | -2.73 (-25.75 – 20.29) | 0.815 | 0.03 (-0.27 – 0.34) | 0.829 |
| Yes | TC | -15.68 (-40.39 – 9.03) | 0.212 | 13.89 (-11.64 – 39.42) | 0.284 | 0.23 (-0.10 – 0.49) | 0.171 |
| No | CC | 22.17 (-46.95 – 91.29) | 0.527 | 47.29 (-24.12 – 118.71) | 0.193 | -0.13 (-0.15 – 0.53) | 0.775 |
| Yes | CC | -0.24 (-49.45 – 48.96) | 0.992 | -10.08 (-60.92 – 40.76) | 0.696 | -1.01 (-1.68 – 0.16) | 0.210 |
| Diabetes | *IDE_9* | **CSF**^a^ |  | **WMH/TTV** |  | **LVV**^a^ |  |
| No | TT | Ref. |  | Ref. |  | Ref. |  |
| Yes | TT | 17.60 (-3.94 – 39.14) | 0.109 | 2.81 (-4.44 – 10.07) | 0.445 | 0.05 (-1.31 – 2.31) | 0.585 |
| No | TC | 10.50 (-15.50 – 36.48) | 0.427 | -3.15 (-11.84 – 5.54) | 0.475 | -0.06 (-0.28 – 0.15) | 0.562 |
| Yes | TC | 1.79 (-27.03 – 30.61) | 0.903 | -3.57 (-13.42 – 6.27) | 0.475 | -0.17 (-0.41 – 0.07) | 0.156 |
| No | CC | -69.46 (-150.1 – 11.14) | 0.147 | -7.39 (-34.36 – 19.57) | 0.589 | -0.72 (-1.39 – 0.05) | 0.245 |
| Yes | CC | 10.32 (-47.10 – 67.71) | 0.723 | 8.43 (-10.75 – 27.63) | 0.387 | 0.37 (-0.11 – 0.84) | 0.132 |

^a^These regions are adjusted for intracranial volume (ICV), age, sex and education.
