# Supplementary material for: HHEX_23 AA Genotype Exacerbates Effect of Diabetes on Dementia and Alzheimer Disease: A Population-Based Longitudinal Study
Source: PLoS Med. 2015 Jul 14;12(7):e1001853. doi: 10.1371/journal.pmed.1001853 (PMC4501827; doi:10.1371/journal.pmed.1001853)
Supplement: S4 Table — (DOCX) [file pmed.1001853.s005.docx]

**S4 Table. Crude and multi-adjusted odds ratios (ORs) and 95% confidence intervals (95% CIs) of the relation of *IDE_9* to diabetes in the Kungsholmen Project and SNAC-K study.**

| ***IDE_9*** | **The Kungsholmen Project** | | |  | **The SNAC-K study** | | |
| --- | --- | --- | --- | --- | --- | --- | --- |
|  | *n*^a^ | Diabetes | |  | *n*^a^ | Diabetes | |
|  |  | OR (95% CI)^b^ | OR (95% CI)^c^ | |  | OR (95% CI)^b^ | OR (95% CI)^c^ |
| TT 40/599 | | 1.00 (Ref.) | 1.00 (Ref.) |  | 129/919 1.00 (Ref.) | | 1.00 (Ref.) |
| TC 11/232 | | 1.06 (0.63–1.81) | 1.05 (0.62–1.90) |  | 57/129 | 1.26 (0.82–2.71) | 1.20 (0.69–3.12) |
| CC 1/22 | | 0.88 (0.64–2.59) | 0.85 (0.54–2.71) |  | 7/38 | 0.92 (0.81–3.23) | 0.88 (0.74–3.77) |
| Phenotypes | | |  |  |  |  |  |
| T 91/1430 0.83 (0.45–1.51) | | | 0.82 (0.42–1.49) |  | 315/1967 0.60 (0.53–1.65) | | 0.71 (0.62–1.75) |
| C 13/274 | | 1.46 (0.92–5.13) | 1.31 (0.86–5.53) |  | 71/205 0.81 (0.66–3.29) | | 0.80 (0.73–4.11) |
| C allele 287 | | 0.98 (0.91–1.96) | |  | 276 0.76 (0.69–1.38) | | |

^a^Number of people with/without diabetes.

^b^Crude OR.

^c^Adjusted for age, sex, education, stroke, heart disease, SBP, DBP, BMI, and *APOE* ε4 status.
