# Supplementary material for: HHEX_23 AA Genotype Exacerbates Effect of Diabetes on Dementia and Alzheimer Disease: A Population-Based Longitudinal Study
Source: PLoS Med. 2015 Jul 14;12(7):e1001853. doi: 10.1371/journal.pmed.1001853 (PMC4501827; doi:10.1371/journal.pmed.1001853)
Supplement: S5 Table — (DOCX) [file pmed.1001853.s006.docx]

**S5 Table. Basic-adjusted hazard ratio (HR) with 95% confidence interval (CI) of dementia and Alzheimer’s disease (AD) related to diabetes, *HHEX_23*, *IDE_9* and *APOE* in the Kungsholmen project and SNAC-K study.**

| **Exposure** | **The Kungsholmen project** | | |  | **The SNAC-K study** | | | |
| --- | --- | --- | --- | --- | --- | --- | --- | --- |
|  | *n* | Dementia  (*n* = 358) | AD  (*n* = 271) |  | | *n* | Dementia  (*n* = 166) | AD  (*n* = 121) |
|  |  | HR (95% CI)^a^ | HR (95% CI)^a^ |  |  |  | HR (95% CI)^a^ | HR (95% CI)^a^ |
| **Diabetes/prediabetes** | | |  |  | |  |  |  |
| No | 856 | 1.00 (Ref.) | 1.00 (Ref.) |  | | 1335 | 1.00 (Ref.) | 1.00 (Ref.) |
| Yes | 114 | 1.76 (1.23–2.29) | 1.69 (1.07–2.38) |  | | 725 | 1.71 (1.07–2.77) | 1.66 (1.03–2.28) |
| ***HHEX_23*** |  |  | |  | |  |  |  |
| GG | 250 | 1.00 (Ref.) | 1.00 (Ref.) |  | | 502 | 1.00 (Ref.) | 1.00 (Ref.) |
| AG | 458 | 0.97 (0.76–1.19) | 1.02 (0.73–1.41) |  | | 1024 | 0.89 (0.39–1.24) | 1.04 (0.68–1.41) |
| AA | 262 | 1.05 (0.77–1.37) | 0.95 (0.72–1.42) |  | | 534 | 0.94 (0.38–1.34) | 0.98 (0.75–1.38) |
| Phenotype |  |  |  |  | |  |  |  |
| G | 708 | 1.03 (0.81–1.29) | 1.01 (0.79–1.36) |  | | 1526 | 0.91 (0.42–2.15) | 0.93 (0.66–2.18) |
| A | 720 | 1.02 (0.83–1.57) | 0.98 (0.79–1.57) |  | | 1558 | 1.01 (0.43–2.07) | 1.00 (0.70–2.29) |
| A allele | 982 | 1.44 (0.88–1.62) | 1.39 (0.85–1.70) |  | | 2092 | 1.52 (0.73–2.10) | 1.47 (0.81–2.12) |
| ***IDE_9*** |  |  |  |  | |  |  |  |
| TT | 674 | 1.00 (Ref.) | 1.00 (Ref.) |  | | 1408 | 1.00 (Ref.) | 1.00 (Ref.) |
| TC | 272 | 1.05 (0.84–1.30) | 1.01 (0.79–1.28) |  | | 586 | 1.09 (0.87–1.39) | 1.09 (0.88–1.41) |
| CC | 24 | 0.69 (0.32–1.53) | 0.69 (0.31–1.56) |  | | 66 | 0.47 (0.16–1.39) | 0.46 (0.16–1.43) |
| Phenotype |  |  |  |  | |  |  |  |
| T | 976 | 1.02 (0.78–1.19) | 1.01 (0.78–1.29) |  | | 1994 | 0.92 (0.41–2.22) | 0.97 (0.69–1.48) |
| C | 296 | 0.95 (0.76–1.24) | 0.94 (0.75–1.27) |  | | 652 | 0.99 (0.53–2.19) | 1.00 (0.72–1.57) |
| C allele | 320 | 1.01 (0.82–1.50) | 0.96 (0.76–1.46) |  | | 718 | 0.97 (0.48–2.24) | 1.02 (0.71–1.34) |
| ***APOE*** |  |  |  |  | |  |  |  |
| No ε4 | 706 | 1.00 (Ref.) | 1.00 (Ref.) |  | | 1487 | 1.00 (Ref.) | 1.00 (Ref.) |
| Any ε4 | 264 | 1.35 (1.02–1.75) | 1.44 (1.05–1.94) |  | | 573 | 1.48 (1.17–2.60) | 1.58 (1.13–1.99) |

^a^Adjusted for age, sex, and education.
