# Supplementary material for: HHEX_23 AA Genotype Exacerbates Effect of Diabetes on Dementia and Alzheimer Disease: A Population-Based Longitudinal Study
Source: PLoS Med. 2015 Jul 14;12(7):e1001853. doi: 10.1371/journal.pmed.1001853 (PMC4501827; doi:10.1371/journal.pmed.1001853)
Supplement: S6 Table — (DOCX) [file pmed.1001853.s007.docx]

**S6 Table. Basic-adjusted hazard ratios (HRs) with confidence intervals (CIs) of dementia and Alzheimer’s disease (AD) related to diabetes (including prediabetes) by *HHEX-23* genotypes in the Kungsholmen project and SNAC-K study.**

| **Joint exposure** | |  | **The Kungsholmen project** | | |  | **The SNAC–K study** | | |
| --- | --- | --- | --- | --- | --- | --- | --- | --- | --- |
| Diabetes | *HHEX-23* |  | *n* | **Dementia** (*n* = 358) | **AD** (*n* = 271) |  | *n* | **Dementia** (*n* = 166) | **AD** (*n* = 121) |
|  |  |  |  | *n* HR (95% CI)^a^ | *n*  HR (95% CI)^a^ |  |  | *n* HR (95% CI)^a^ | *n*  HR (95% CI)^a^ |
| No | GG |  | 224 | 88 1.00 (Ref.) | 71 1.00 (Ref.) |  | 335 | 26 1.00 (Ref.) | 19 1.00 (Ref.) |
| Yes | GG |  | 26 | 11 0.92 (0.67–2.81) | 7 0.89 (0.64–3.09) |  | 167 | 18 0.76 (0.45–2.38) | 12 0.77 (0.36–2.49) |
| No | AG |  | 408 | 140 0.81 (0.63–1.04) | 107 0.85 (0.64–1.13) |  | 664 | 52 0.89 (0.51–1.19) | 43 0.85 (0.39–1.64) |
| Yes | AG |  | 50 | 13 1.77 (0.74–3.76) | 9 1.82 (0.85–3.83) |  | 360 | 29 1.80 (0.83–4.36) | 20 1.68 (0.69–4.42) |
| No | AA |  | 224 | 95 0.93 (0.71–1.23) | 69 0.90 (0.65–1.25) |  | 336 | 24 0.82 (0.55–1.11) | 16 0.75 (0.43–1.18) |
| Yes | AA |  | 38 | 11 5.63 (2.35–6.92) | 8 5.02 (2.43–7.96) |  | 198 | 17 4.92 (1.93–5.59) | 11 4.65 (1.74–6.02) |

^a^Adjusted for age, sex, and education.
